# DL-PCMNet: Distributed Learning Enabled Parallel Convolutional Memory Network for Skin Cancer Classification with Dermatoscopic Images

**DOI:** 10.3390/diagnostics16020359

**Published:** 2026-01-22

**Authors:** Afnan M. Alhassan, Nouf I. Altmami

**Affiliations:** Department of Computer Science, College of Computing and Information Technology, Shaqra University, Shaqra 11961, Saudi Arabia

**Keywords:** skin cancer classification, deep learning, medical imaging, distributed learning, dermatoscopic image

## Abstract

**Background/Objectives**: Globally, one of the most dreadful and rapidly spreading illnesses is skin cancer, and it is acknowledged as a lethal form of cancer due to the abnormal growth of skin cells. Mostly, classifying and diagnosing the types of skin lesions is complex, and recognizing tumors from dermoscopic images remains challenging. The existing methods have limitations like insufficient datasets, computational complexity, class imbalance issues, and poor classification performance. **Methods**: This research presents a method named the Distributed Learning enabled Parallel Convolutional Memory Network (DL-PCMNet) model to effectively classify skin cancer by overcoming the existing limitations. Hence, the proposed DL-PCMNet model utilizes a distributed learning framework to provide greater flexibility during the learning process, and it increases the reliability of the model. Moreover, the model integrates the Convolutional Neural Network (CNN) and Long Short-Term Memory model (LSTM) in a parallel distribution, which enhances robustness and accuracy by capturing the information of long-term dependencies. Furthermore, the utilization of advanced preprocessing and feature extraction techniques increases the accuracy of classification. **Results**: The evaluation results exhibit an accuracy of 97.28%, precision of 97.30%, sensitivity of 97.17%, and specificity of 97.72% at 90% of training by using the ISIC 2019 skin lesion dataset, respectively. **Conclusions**: Specifically, the proposed DL-PCMNet model achieved efficient and accurate skin cancer classification compared with other existing models.

## 1. Introduction

The abnormal cells produced in our body can lead to cancer, and they reproduce uncontrollably by growing to different parts of the body. Typically, cancer can be classified into various types, such as lung cancer, liver cancer, breast cancer, skin cancer, and so on, which are the main causes of human death. Moreover, the most widely spreading cancer is skin cancer, which also causes death [1], and it is considered the world’s fastest spreading type of cancer due to the uncontrollable growth of skin cells [2]. Generally, melanoma and benign tumors occur due to the growth of skin cells [3]. Malignant tumors grow and quickly spread to other body parts [4,5], whereas benign tumors usually do not spread to other body parts, but they form in the skin [6]. The abnormal increase in melanocytic cells leads to the growth of skin tumors, and they are a life-threatening and harmful disease if left untreated [7]. Some of the frequent varieties of skin cancers include dermato-fibroma, basal cell carcinoma, melanocytic-nevus, squamous cell carcinoma, vascular-lesion, and benign-keratosis, which are different types of skin conditions [8]. Factors like ultraviolet (UV) rays trigger and penetrate the skin cells’ abnormal condition, which leads to several types of tumors, such as melanoma, which is associated with UV exposure [9].

In the early stage, diagnosing the cancer can increase the rate of survival, but it is difficult to identify the cancer disease with the naked eye because it is subjective [10]. To prevent any type of skin cancer, it is important to identify and evaluate it in the early stage [4]. In current clinical practice, dermoscopy is used to diagnose skin cancer and identify various types of skin cancer, because clinical visual results can be unreliable [11]. By exploiting a specialized device to illuminate and magnify the skin, physicians can see the skin’s surface by using dermoscopy techniques. [12]. Typically, by utilizing a variety of different models, skin cancer diagnosis is developed, and the treatment is improved by introducing effective approaches [13]. In traditional approaches, effective Machine Learning (ML) methods are incorporated to extract skin images with high-level characteristics, and they are classified based on these features. In dermatology, the advancement of ML technology continues to enhance speed, accuracy, and scalability [14]. Through their advancement, deep learning (DL) approaches have shown numerous benefits over melanoma detection techniques, and these methods do not support the interpretability of models related to pathological signs of features in the melanoma images [15].

DL and computer vision algorithms are potentially leveraged for the enhancement of the accuracy and speed of skin cancer diagnosis, which enables personalized and timely intervention in cancer treatment [16]. In skin cancer classification, the DL technique is used to categorize the skin cancer lesions that have outperformed manual specialists [17]. Moreover, the CNNs exploit low-resolution images to minimize the number of parameters and calculations in the network, which leads to a loss of important features [11]. However, the labeled dermoscopic images are analyzed widely to identify the characteristics, such as color distributions and visual patterns that help to identify different skin type conditions [8]. Most of the existing methods in skin cancer classification have faced several challenges, such as complexity issues, dataset limitations, and poor reliability and interpretability. Hence, the DL approaches are utilized in this research to effectively identify different varieties of skin cancer.

This research presents the DL-PCMNet model, which integrates a distributed learning framework with a DL approach to classify the skin cancer types. However, the model utilizes a large dataset and advanced preprocessing and feature extraction techniques to learn and extract significant features that help to differentiate cancer characteristics. Thereafter, extracted features are applied to the proposed DL-PCMNet model, and it is trained well to classify skin cancer. The major contribution of the proposed research is explained below.

Distributed Learning enabled Parallel Convolutional Memory Network model (DL-PCMNet): The incorporation of distributed learning enables a model to be trained over multiple devices, which enhances the training and inference time. Furthermore, the integration of CNN and LSTM in the parallel distribution captures spatial and temporal features, which often leads to accurate skin cancer classification.

This paper is organized into the subsequent sections: Section 2 reviews the existing works with challenges, the detailed flow and system architecture are presented in Section 3, an in-depth analysis of the proposed architecture is explored in Section 4, and Section 5 presents the results with different analyses and implications. Lastly, future work of the research is explained in Section 6.

## 2. Literature Review

Some of the existing literature papers are analyzed, and their limitations are briefly described below. Saeed, M. et al. [1] developed a CNN model that exploits Enhanced Super-Resolution Generative Adversarial Networks and Generative Adversarial Networks. Here, the hybrid Visual Geometry Group-Support Vector Machine (VGG-SVM) exhibits higher accuracy, and the model classifies skin cancer with different types from dermoscopic images through augmentation and pre-processing steps. Typically, the augmentation technique is employed for increasing the size and variety of the dataset by applying different transformations like flips and rotations. The model achieves higher classification accuracy, and it accurately detects skin cancer. Moreover, the primary challenge in the model was scalability and computational complexity. Arshed, M.A. et al. [2] designed an approach named an off-the-shelf Vision Transformer (ViT) for identifying skin cancer. The model uses a technique of data augmentation to increase the dataset size, and it applies alterations to the data. Moreover, the method integrates fine-tuning and transfer learning to achieve higher results. The ViT exploited in the model improves the classification results, and the method comprises transformer layers, hidden units, and trainable parameters. The model had overfitting issues, which were considered the main challenge.

Keerthana, D. et al. [3] developed two hybrid CNN models by employing MobileNet and DenseNet-201 with an SVM classifier. However, the features are extracted by performing classification and passed into the SVM classifier. Here, the SVM is used to separate the data points, and it classifies the new observations based on predictive methods. The model utilizes multiple pre-trained models for classifying skin cancer, and they achieve the highest accuracy. The model faced issues such as performance degradation, high computational costs, and risk while handling large amounts of data. Gururaj, H.L. et al. [4] explained an advanced deep learning-based CNN model that classifies skin cancer. Generally, the method of oversampling was used to balance the dataset, and the majority of samples were reduced to obtain the distribution of classes. However, the performance of the model was validated using a dataset, and the training of the model took place by utilizing transfer learning methods like ResNet50 and DenseNet169. Moreover, the model achieves better performance, but it faces issues like poor generalization and reduced interpretability.

Mridha, K. et al. [5] developed a DL-based optimized CNN model with Explainable Artificial Intelligence (XAI) to diagnose the type of skin cancer. At first, the model collects input data from the dataset, and pre-processing is carried out by performing normalizing, resizing, and rescaling. After that, features are obtained, and a classifier is constructed by utilizing optimization algorithms. An augmentation technique was exploited for increasing the number of training samples, and the model attained high classification accuracy. Magdy, A. et al. [6] explained two approaches, such as using k-nearest neighbor (KNN) as a classifier, and AlexNet with the gray wolf optimizer (GWO). However, the performance of the methods is compared with DL and ML techniques, and a dataset was used to validate the model. The model requires high-quality medical data for training, and it faced class imbalance problems, which were considered the main challenge.

Gallazzi, M. et al. [7] suggested a Swin Transformer (ST)-based deep neural network with a self-attention mechanism for diagnosing the types of skin cancer disease. However, the method utilizes transformer models that accurately capture the spatial dependencies across the region of images without relying on pre-processing. Generally, the efficiency of the model was measured with different trained models, and the model achieves high performance with improved generalization capabilities. Hosny, M. et al. [8] explained an efficient dual attention mechanism based on the ResNet50 (EDA-ResNet50) model. Typically, the model captures essential features across the lesion areas, and it incorporates multi-scale feature representation blocks. However, the model integrates explainability to improve the transparency in the process of decision-making. The model achieves better classification performance, but it fails to integrate effective attention mechanisms and advanced deep learning techniques.

### 2.1. Challenges

The drawbacks faced by the traditional methods are briefly discussed below:

➢ The method VGG-SVM in [1] faced high computational complexity issues, and it restricts its applicability to clinical applications;
➢ The model suggested in [3] had class imbalance issues in the dataset because it needed a vast amount of medical image data for effective training;
➢ The model encountered in [4] is computationally expensive, and evaluating the model can utilize additional resources;
➢ The model in [5] cannot integrate attention or transformer mechanisms, and in [6], the model faces high class imbalance issues;
➢ The model suggested in [8] achieves better performance, but it limits its interpretability in clinical applications due to a lack of transparency.

### 2.2. Problem Statement

The unnecessary growth of melanocytic cells leads to skin cancer, and they globally increase human death rates. Skin cancer is very dangerous because it can affect any part of the human body. However, diagnosing and identifying skin cancer in the early stage can improve the outcomes of treatment and lower the death rates. The traditional approaches employed for skin cancer detection in the early stage faced significant limitations, such as overfitting, insufficient datasets, limited performance, and computational complexity. Here, these existing difficulties are tackled by the proposed model, named DL-PCMNet, which accurately classifies different kinds of skin cancer. Moreover, the datasets utilized for this research are the ISIC 2019 Skin Lesion dataset [18] and the Skin Cancer MNIST: HAM10000 dataset [19], respectively. Let us assume that some of the dermatoscopic images are gathered from different populations, and they are stored in several modalities, which are mathematically expressed as

(1) $$G={\left\{P_{i},Q_{i}\right\}}_{i\in 1\ldots n}$$

where G implies the dataset, $P_{i}$ represents the total dermatoscopic images, $Q_{i}$ denotes the type of diagnostic category for total images, and here, $P_{i}\in {ℜ}^{C\times I\times W}$ denotes the height, width, and channels of each dermatoscopic image. After collecting the images from the dataset, the image processing technique is utilized to remove unwanted noise from the images. Thereafter, extracted features are applied to the proposed model. Here, the proposed DL-PCMNet model achieves good classification performance, and its effectiveness is analyzed by reducing the error rate using Categorical Cross-Entropy (CCE). The loss function is represented as

(2) $$L_{CCE}=-\sum_{i=1}^{n}Q_{i}\left(\log\left({\hat{Q}}_{i}\right)\right)$$

where n indicates the total number of classes, $Q_{i}$ denotes the actual label for $i^{th}$ class, and ${\hat{Q}}_{i}$ indicates the predicted label. Here, the actual label values for the ISIC 2019 Skin Lesion dataset [18] are represented as

(3) $$Q_{i}=\left\{\begin{array}{l} Actinic\;\ker atosis\\ \mathrm{Benign}\;\mathrm{keratosis}\\ \mathrm{Melanoma}\\ \mathrm{Melanocytic}\;\mathrm{nevus}\\ \mathrm{Dermatofibroma}\\ \mathrm{Squamous}\;\mathrm{cell}\;\mathrm{carcinoma}\\ Basal\;cell\;carcinoma\\ \mathrm{Vascular}\;\mathrm{lesion} \end{array}\right.$$

The actual label values for the Skin Cancer MNIST: HAM10000 dataset [19] are expressed as follows:

(4) $$Q_{i}=\left\{\begin{array}{l} \mathrm{Vascular}\;\mathrm{lesion}\\ \mathrm{Melanoma}\\ \mathrm{Dermatofibroma}\\ \mathrm{Benign}\;\mathrm{keratosis}\\ \mathrm{Basal}\;\mathrm{cell}\;\mathrm{carcinoma}\\ \mathrm{Melanocytic}\;\mathrm{nevus}\\ \mathrm{Actinic}\;\mathrm{keratosis} \end{array}\right.$$

## 3. System Model

**Figure 1:**
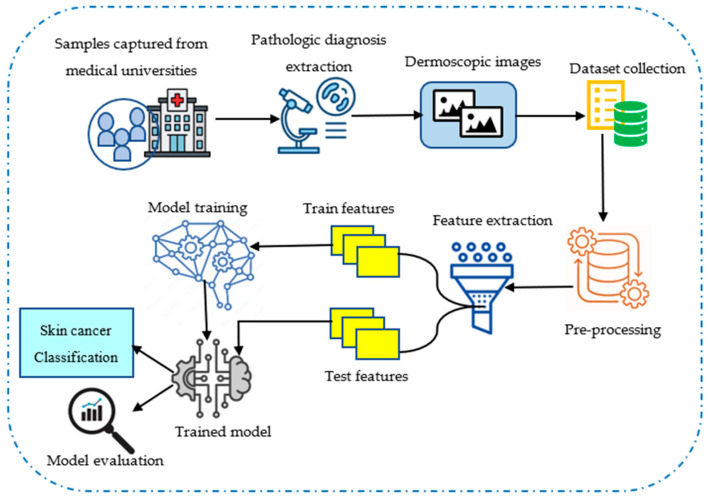
System model for skin cancer classification.

The most common variety of cancer that leads to death is skin cancer, and it spreads due to the uncontrollable growth of abnormal skin cells. However, skin cancer is recognized as a dreadful disease because it poses a significant threat to individuals. Initially, the dataset contains dermatoscopic image samples that are obtained from various medical universities. After collecting the images, they are applied to remove noise from the images in the pre-processing phase. Next, the pre-processed data is passed to feature extraction, where important high-level features are extracted to improve the model performance. Thereafter, informative features are given to the proposed method, and it is trained well to perform skin cancer classification. The detailed system model is illustrated in Figure 1.

## 4. Skin Cancer Classification Using Distributed Learning Enabled Parallel Convolutional Memory Network

**Figure 2:**
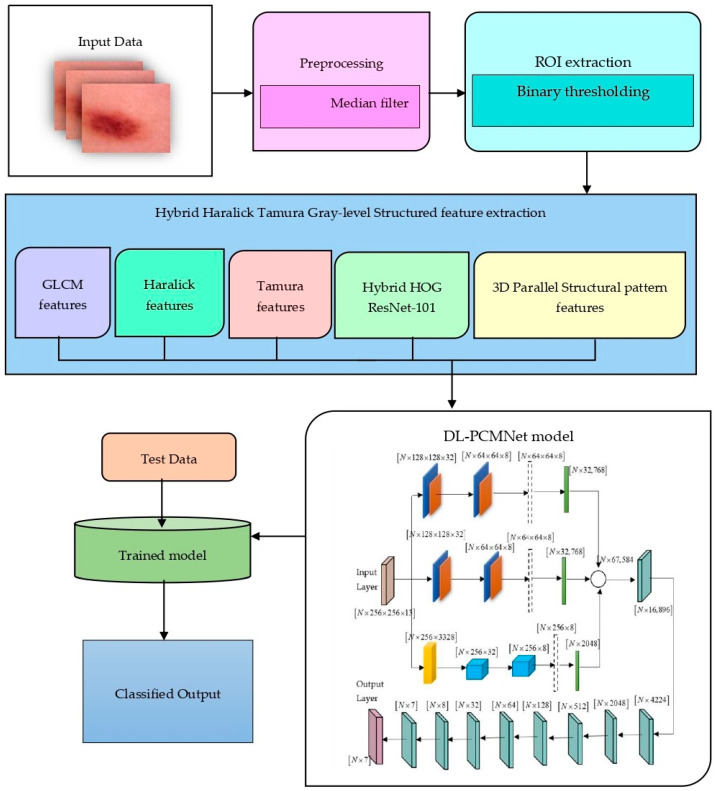
Workflow of the proposed DL-PCMNet model for skin cancer classification.

Skin cancer is a widely recognized malignant melanoma subtype that is characterized by abnormal melanocyte cell growth. The existing models for identifying skin cancer have faced certain limitations, such as complexity, limited datasets, and poor generalizability. To overcome these limitations, this research utilized advanced preprocessing and feature extraction techniques to enhance the image quality and extract significant features. The research focuses on classifying types of skin cancer disease at an early stage by exploiting the DL-PCMNet model effectively. At first, dermatoscopic images are collected as input from the two datasets, and they are further passed to the image pre-processing stage. Here, pre-processing and ROI extraction are applied to extract informative regions from the original images. After extracting the regions, the output image undergoes the feature extraction phase, where the important and meaningful features are extracted from the images. Various feature extraction approaches, such as GLCM (Gray-level Co-occurrence Matrix) features, Haralick features, Tamura features, Hybrid Hog ResNet-101 features, and 3D-parallel structured pattern features, are employed in this phase. Thereafter, the extracted features are concatenated and directed into the proposed DL-PCMNet model for effective training. Moreover, the proposed model incorporated distributed learning, where the mirrored strategy is utilized to create a replica of the model in multiple devices. Hence, the model is trained on multiple devices, and the output is averaged by all the reduction algorithms to enhance the performance of the model. Here, the CNN and LSTMs are employed using parallel distribution to extract spatial and temporal features simultaneously, which improves the classification process. Hence, the trained model is tested with data and validated for the skin cancer classification performance. Overall, the DL-PCMNet model accurately classifies the type of skin cancer into multiple classes and improves the training and inference time. The structure of the DL-PCMNet model is visualized in Figure 2.

### 4.1. Collection of Input Data

Initially, dermoscopic images are acquired from the ISIC 2019 Skin Lesion [18] and the Skin Cancer MNIST: HAM10000 [19] datasets, and these datasets have unique dimensions. Let us consider the dataset G with n number of image samples; it is expressed as

(5) $$G=\left\{P_{1},P_{2},\ldots P_{i}\ldots ,P_{n}\right\}$$

Here, the dataset is denoted as G, n indicates the number of images with the dimension $\left[N\times 450\times 600\times 3\right]$, and $P_{i}$ implies the $i^{th}$ dermatoscopic image.

### 4.2. Data Pre-Processing and ROI Extraction

Image pre-processing becomes an essential step because the quality of original images is improved and the noises are reduced from the images. Initially, a median filter is utilized in the pre-processing phase, and it is applied to the input image $P_{i}$, which is obtained from the dataset. However, a median filter is a nonlinear filter or digital filter that exchanges the value of each pixel with the median value of neighboring pixels to reduce the noise. The main purpose of this filter is to reduce the noise from the images by preserving the edges with important information. After applying the median filter, the output value is indicated as $P_{i}^{*}$, which has the dimension $\left[N\times 450\times 600\times 3\right]$. Thereafter, the extraction of ROI is performed on the obtained $P_{i}^{*}$ image, where the image is initially converted into a grayscale form. In a grayscale image, the color information of each pixel is removed and replaced with single intensity values, which range from 0 to 255. Furthermore, an image with grayscale is transformed into a binary image by employing binary thresholding. In the binary thresholding process, the values of each pixel’s intensity are analyzed with the threshold value. When the pixels have an intensity value higher than the threshold, the maximum value ‘255’ is set, whereas the minimum value ‘0’ is set for pixels with an intensity value less than or equal to the threshold value. Furthermore, contour detection is applied to the binary image, where it effectively extracts top-level boundaries and regions from the image to define the shape of distinct objects. Here, each counter is typically denoted as a NumPy array of $\left(x,y\right)$ coordinates with its boundary points, and the skin region is extracted based on the boundary. Next, the output of each image obtained from the counter detection process varies with size; hence, they are resized to a common dimension $\left[N\times 256\times 256\right]$. Finally, the output obtained from the ROI extraction phase can be represented as S, which has the dimension $\left[N\times 256\times 256\times 1\right]$.

### 4.3. Feature Extraction Using Hybrid Haralick Tamura Gray-Level Structured Features (H^2^TGS)

The process of feature extraction helps to identify and extract meaningful and relevant information from the input skin images. However, the H^2^TGS approach is employed to extract important and informative features from the input images, such that the H^2^TGS-based feature model is the combination of different features, such as GLCM features, Haralick features, Tamura features, Hybrid Hog ResNet-101 features, and a 3D-parallel structured pattern feature, respectively. Here, the H^2^TGS features are exploited to extract the prominent information of images, and they enhance the model’s accuracy, speed, and efficiency by identifying the attributes such as patterns, textures, and shapes of the images.

#### 4.3.1. Gray-Level Co-Occurrence Matrix Feature

The GLCM is a statistical method of examining texture features of images by utilizing the gray-level transition of the image between neighboring pixels [20]. However, the GLCM consists of various rows and columns, which are equal to the number of gray levels in the image. Here, the GLCM feature is mainly utilized for determining the texture of an image, and it captures spatial relationships between the pixels, which leads to high accuracy in image classification. The entries in GLCM denote the number of pairs of pixels with the same level of brightness, separated by angle and distance. Let us assume the co-occurrence matrix is denoted as $S_{b,c}$ and the matrix size is represented as R×R. The frequency is represented for each element $\left(b,c\right)$, in which the gray level pixel b is related to the number of pixels with a gray level c. The relation of the neighbor pixel c and the reference pixel b is represented in GLCM across various orientations. Here, the GLCM takes the extracted ROI grayscale image S as input data, and the output of the GLCM is a co-occurrence matrix in which the features are extracted based on statistical feature parameters, such as homogeneity, contrast, entropy, dissimilarity, and Angular Second Moment (ASM). Table 1 depicts the texture features that are obtained from the GLCM.

Finally, the GLCM features are concatenated and indicated as F with the magnitude of $\left[N\times 256\times 256\times 5\right]$:

(6) $$F=\left\{F_{1}\left\|F_{2}\left\|F_{3}\left\|F_{4}\left\|F_{5}\right.\right.\right.\right.\right\}$$

#### 4.3.2. Haralick Features

The texture of an image is described by the Haralick feature because it provides information about different pixel combinations within an image. However, the GLCM is used to calculate the statistical measures that form the Haralick features [21]. Notably, an important objective of the Haralick feature is to capture the spatial relationship between the pixels, and it offers flexibility while capturing texture information under various conditions. The Haralick feature takes the ROI-extracted grayscale image S as input; then, it produces an output that is represented as H. Furthermore, the dimension obtained from the Haralick feature is denoted as $\left[N\times 256\times 256\times 1\right]$.

#### 4.3.3. Tamura Feature

The Tamura features mainly focus on texture analysis that measures and identifies the visual properties of textures such as contrast, directionality, and coarseness. To compute the Tamura feature for each window, the statistical distribution, Fourier spectrum, and autocorrelation function are analyzed separately. The Tamura features improve the discriminative accuracy of textures, and they capture perceptual properties of textures such as contrast, roughness, and directionality. Moreover, these perceptual characteristics are very important for capturing the visual characteristics that provide textures with meaningful representations [22]. The most commonly used Tamura texture features are outlined as follows:

**Contrast feature:** The differences in local intensity that occur inside the textures are measured by the contrast feature. The mathematical equation of the contrast feature is expressed as follows:

(7) $$T_{1}=\sqrt{\sum_{b=0}^{L-1}S\left(b\right)\cdot {\left(b-\mu \right)}^{2}}$$

Here, L indicates the number of gray levels, S(b) denotes the probability of gray level, and μ indicates the mean gray level. The output from the contrast feature is denoted as $T_{1}$ with the magnitude $\left[N\times 256\times 256\times 1\right]$.

**Coarseness characteristic:** The coarseness characteristic measures the roughness of the textures, and it illustrates the repeated patterns that are visible in the texture. The autocorrelation function is expressed as follows:

(8) $$T_{2}=\frac{1}{uv}\sum_{r=0}^{u-1}\sum_{s=0}^{v-1}\left[O\left(r,s\right)-\mu \right]\left[O\left(r+l,s\right)-\mu \right]$$

where l denotes the distance between pixel pairs, O(r,s) represents the intensity of a pixel, and u,v indicates the dimensions of the image. The output from the coarseness characteristic is represented as $T_{2}$, which has the dimension $\left[N\times 256\times 256\times 1\right]$.

**Directionality feature:** In the Tamura feature, the directionality formula measures the sharpness of the peaks in the directional histogram of an image. The equation for the directionality feature is expressed as follows:

(9) $$T_{3}=1-o\cdot H_{g}\sum_{g=1}^{H_{g}}\sum_{\varphi \in \gamma_{g}}{\left(\varphi -\varphi_{g}\right)}^{2}H_{d}\left(\varphi \right)$$

where $H_{g}$ denotes the number of peaks in the histogram, o indicates the normalizing factor, $\varphi_{g}$ is the position of the angle, and $H_{d}(\varphi )$ represents the histogram with quantized direction values. The output from the directionality feature is denoted as $T_{3}$, which has the dimension of $\left[N\times 256\times 256\times 1\right]$.

Overall, the concatenated Tamura features are represented as T, with the magnitude of $\left[N\times 256\times 256\times 3\right]$, which is expressed as

(10) $$T=\left\{T_{1}\left\|T_{2}\left\|T_{3}\right.\right.\right\}$$

#### 4.3.4. Hybrid HOG ResNet-101 Features

In this feature extraction, Histogram of Oriented Gradients (HOG) features and ResNet-101 features are combined to achieve high accuracy, and they help to prevent the problem of vanishing gradient, allowing for effective training of the deep neural network. Moreover, the hybrid models provide greater robustness and generalization capabilities, which help to overcome the issues of overfitting. Initially, the image S is passed as input to the HOG feature descriptor. However, the HOG feature captures the structure and shape of the objects by analyzing the distribution of orientation and gradients within the localized regions of an image [23]. Furthermore, the output obtained from the HOG descriptor is a feature vector, and its size is represented as $\left[N\times 256\times 256\times 1\right]$. Here, the output acquired from the HOG descriptor is resized into three channels with dimensions $\left[N\times 256\times 256\times 3\right]$, before being given to the ResNet-101 features. Finally, the dimension is resized into the dimension of $\left[N\times 256\times 256\times 1\right]$, and the output of the hybrid HOG ResNet-101 feature is denoted as R.

#### 4.3.5. Three-Dimensional Parallel Structural Pattern Features

The 3D parallel structural pattern is obtained by the extraction of textural patterns such as Local Ternary Pattern (LTP), Local Binary Pattern (LBP), and Local Gradient Pattern (LGP). The main benefits of 3D parallel structural patterns are that they enhance performance, improve computational efficiency, and enable faster execution time by capturing multi-scale information. At first, the output S from the ROI extracted region is fed in parallel into LTP, LBP, and LGP, respectively. The texture operator LBP is mainly utilized for measuring local contrast of an image [24]. The expression for the LBP operation is denoted as

(11) $$LBP_{U,V}=\sum_{g=0}^{U-1}E(J_{g}-J_{e})2^{g}$$

(12) $$E(g)=\left\{\begin{array}{l} 1,\;\;if\;g\geq 0\\ 0,\;\;if\;g<0 \end{array}\right.$$

where $J_{e}$ represents the central pixel of the frame, $J_{g}$ indicates the frame’s adjacent pixel, and V denotes the radius. However, output from the operation LBP is denoted as $B_{1}$, and it obtains the dimension $\left[N\times 256\times 256\times 1\right]$. Likewise, the textural feature LTP gives a set of binary values, and it is exceptionally robust to noise. The mathematical expression of LTP is represented as

(13) $$LTP=\left\{\begin{array}{l} 1\;\;if\;J_{g}>J_{e}+K\\ 0\;\;if\;J_{g}>J_{e}-K\\ -1\;\;if\;J_{g}<J_{e}-K \end{array}\right.$$

Here, K indicates the number of clusters. Furthermore, the outcome of the LTP operation is represented as $B_{2}$, which obtains the magnitude $\left[N\times 256\times 256\times 1\right]$. Similarly, the LGP is a feature descriptor employed in image processing to analyze the local texture of images by encoding the relationship between the neighboring pixel and the central pixel based on their gradient [25]. The operation of LGP is mathematically expressed as

(14) $$LGP_{U,V}=\sum_{g=0}^{U-1}h\left(t_{m}-\bar{t}\right)\times 2^{g}$$

where $\bar{g}$ denotes the average of all gradient values and m indicates the index of the neighbor. Furthermore, the outcome acquired from the LGP operation is symbolized as $B_{3}$, with the magnitude $\left[N\times 256\times 256\times 1\right]$. Overall, the output values are concatenated as $B_{1}$, $B_{2}$, and $B_{3}$, and represented as B with the dimension $\left[N\times 256\times 256\times 3\right]$.

(15) $$B=\left\{B_{1}\left\|B_{2}\left\|B_{3}\right.\right.\right\}$$

Finally, the extracted features received from the GLCM features, Haralick features, Tamura features, Hybrid Hog ResNet-101 features, and a 3D-parallel structured pattern feature are concatenated and generate the H^2^TGS features, and they are expressed as

(16) $$Z=\left\{F\left\|H\left\|T\left\|R\left\|B\right.\right.\right.\right.\right\}$$

where Z denotes the H^2^TGS features with the dimension $\left[N\times 256\times 256\times 13\right]$, and it is further applied to the proposed model.

### 4.4. Distributed Learning Enabled Parallel Convolutional Memory Network Model

**Figure 3:**
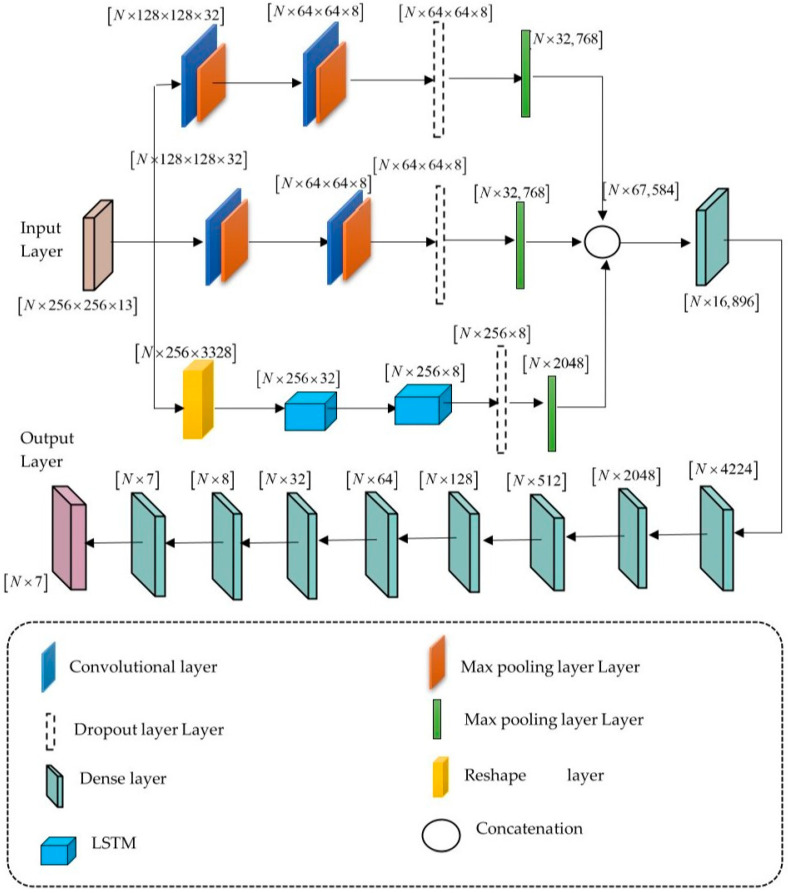
Architecture of the proposed DL-PCMNet model.

The proposed DL-PCMNet model is utilized accurately to perform the classification of skin cancer disease. Here, the model is derived through the combination of a CNN and an LSTM network, with a distributed learning mechanism. In this research, the proposed model executes the distributed learning using a mirrored strategy, where the model is copied over multiple devices within the model to enhance the classification process. The CNN and LSTM networks are integrated using the parallel mechanism. Furthermore, the utilization of a distributed learning and a parallel mechanism minimizes the overall training time of the model and increases the performance. However, the distributed learning approach is useful for complex models with large datasets, which may take some time to train and generate better results on unseen data. The benefits of distributed learning improve classification accuracy and scalability, with reductions in training time leading to better generalizability. The architecture of the proposed DL-PCMNet model is visualized in Figure 3.

Initially, the DL-PCMNet model takes the input data as extracted features Z with dimension $\left[N\times 256\times 256\times 13\right]$, and it is applied to the LSTM block and the convolutional blocks separately. The model utilizes a parallel distribution mechanism to enhance the computational speed, performance, and robustness by leveraging parallel processing capabilities. The detailed structure of the convolutional block is briefly discussed below. Here, the convolutional block is composed of maxpooling layers, convolutional layers, flatten layers, and dropout layers, respectively. At first, the input features with magnitude $\left[N\times 256\times 256\times 13\right]$ are applied to the convolutional layer, although the input spatial features are obtained through convolutional layers with several filters of shapes and sizes [20]. Moreover, the mathematical equation of the convolutional layer is denoted as

(17) $$A=R_{a}(d+\sum_{j=1}^{J}M[c]*y[c])$$

where A denotes the outcome of the convolutional layer, d is the bias term, y[c] represents kernel input, $R_{a}$ indicates the activation function, M[c] indicates the kernel weight, J implies total input channels, and * denotes operations of convolution. Thereafter, the convolutional layer’s output is passed to the maxpooling layer. Generally, the maxpooling layer is employed here to minimize the computational complexity issues, the feature map’s size, and the number of parameters. Moreover, it enables the proposed DL-PCMNet model to achieve robust and high performance. Thereafter, the outcome of the maxpooling layer with magnitude $\left[N\times 128\times 128\times 32\right]$ is forwarded again to the convolutional layer, where the same operations are executed. Next, the convolutional layer’s output is applied to the maxpooling layer. The maxpooling layer’s output with magnitude $\left[N\times 64\times 64\times 8\right]$ is then forwarded to the dropout layer. Here, the dropout layer is exploited to prevent overfitting issues, and it allows the network for learning robust and generalizable features. The dropout layer outcome with dimension $\left[N\times 64\times 64\times 8\right]$ is further passed to the flatten layer. Typically, the flatten layer is responsible for transforming the output data into a single vector. Thereafter, the output obtained from the flatten layer is indicated as M, which has the dimension $\left[N\times 32768\right]$.

Likewise, other convolutional block also performs the same above operations, and the output from the flatten layer can be indicated as $M^{\prime }$ with the magnitude $\left[N\times 32768\right]$. Consequently, the input feature Z is applied to the LSTM block, where the structure of the LSTM block is briefly discussed below. At first, the input feature with magnitude $\left[N\times 256\times 256\times 13\right]$ is resized, and the resized dimension $\left[N\times 256\times 3328\right]$ is further passed to the LSTM block. The LSTM block is developed to tackle the disappearing gradient issue in conventional RNNs, and it captures dependencies within the sequential data. The architecture of the LSTM consists of forget, input, output, and cell gates. The input gate aims to control the data stored, and the output gate determines the subsequent state. Let us consider the series of images as $\left(X_{1},X_{2},X_{3},\ldots ,X_{e}\right)$, and at time k, the cell state and hidden state are denoted as $a_{k}$ and $w_{k}$, respectively [26]. The expressions for the LSTM block are mathematically represented as follows

(18) $$f_{k}=\sigma \left(N_{q}\cdot \left[y_{k-1},z_{k}\right]+p_{q}\right)$$

(19) $$h_{k}=\sigma \left(N_{b}\cdot \left[y_{k-1},z_{k}\right]+p_{b}\right)$$

(20) $${\tilde{a}}_{k}=\tanh\left(N_{d}\cdot \left[y_{k-1},z_{k}\right]+p_{d}\right)$$

(21) $$a_{k}=f_{k}*a_{k-1}+h_{k*}{\tilde{a}}_{k}$$

where $N_{q},\;N_{b},N_{d}$ denotes the weighted matrices, $p_{q},p_{b},p_{d}$ are the biases of the LSTM, and σ implies the sigmoid function. Furthermore, the output gate and the updated cell states are expressed as

(22) $$o_{k}=\sigma \left(N_{o}\cdot \left[y_{k-1},z_{k}\right]+p_{o}\right)$$

(23) $$w_{k}=o_{k}*\tanh\left(a_{k}\right)$$

where $w_{k}$ denotes the hidden state or output of the LSTM block, which is further forwarded to another LSTM block. Thereafter, the LSTM block’s outcome with magnitude $\left[N\times 256\times 8\right]$ is fed into the dropout layer. Next, the outcome of the dropout layer is forwarded to the flatten layer, which is denoted as Y, and the flattened layer’s dimension is represented as $\left[N\times 2048\right]$, respectively. Here, the output obtained from parallel convolutional blocks and the LSTM block is denoted as M, $M^{\prime }$, and Y, which are further concatenated to obtain the dimension as $\left[N\times 67,584\right]$, which are denoted as

(24) $$N=\left\{M\left\|M^{\prime }\left\|Y\right.\right.\right\}$$

Furthermore, the concatenated output $\left[N\times 67,584\right]$ is obtained, which is passed to the dense layer. In this model, the architecture of the dense block is composed of 8 dense layers, which are dense-0, dense-1, dense-2, dense-3, dense-4, dense-5, dense-6, dense-7, and dense-8, respectively. Here, the process is performed sequentially, where all the layers are connected directly to one another. The concatenated output is passed to dense-0, and the outcome of the dense-0 layer with magnitude $\left[N\times 16,896\right]$ is forwarded into the dense-1 layer. Furthermore, the dense-1 layer’s output with dimension $\left[N\times 4224\right]$ is further passed to the dense-2 layer. Likewise, the outputs with dimensions are forwarded sequentially into all the dense layers. Lastly, the dense layer-8 classifies the output into seven classes, which is indicated as ${\hat{Q}}_{i}$, with the dimension of $\left[N\times 7\right]$, respectively.

## 5. Results and Discussion

This section summarizes the experimental results obtained for the proposed DL-PCMNet model as well as the conventional methods in classifying skin cancer. Moreover, the experimental setup, performance metrics, and dataset description are summarized.

### 5.1. Experimental Setup

The tool employed for the implementation of the DL-PCMNet model is Python 3.7, Intel i7-13770k processor, Windows 11 operating system, 12 GB of GPU memory, 16 GB of RAM, and 128 GB of ROM. Here, the exploitation of two datasets evaluates the DL-PCMNet model’s performance, and libraries such as Keras 2.5, PyTorch 2.1, and TensorFlow 2.5 are implemented for experimentation. Specifically, the proposed model utilizes 90% of the data for training purposes and 10% for testing and validation purposes for each dataset. The DL-PCMNet model’s hyperparameters are described in Table 2.

### 5.2. Dataset Description

In the ISIC 2019 Skin Lesion dataset [18], 25,331 skin lesion images are available, and they are used in dermoscopic image classification with different categories, such as Melanocytic nevus, dermatofibroma, squamous cell carcinoma, melanoma, benign keratosis, and so on. This publicly available dataset is typically used for various types of research, and the images are collected from various medical universities and other resources. A total of 10,015 images from this dataset were utilized for skin cancer classification.

In the Skin Cancer MNIST: HAM10000 dataset [19], pigmented skin lesion images are available, and they are gathered with respect to different populations and stored in several modalities. The dataset contains 10,015 dermoscopic images with different diagnostic categories. The pigmented lesions are intraepithelial carcinoma, basal cell carcinoma, dermatofibroma, actinic keratoses, melanocytic nevi, and so on. Moreover, the dataset contains lesions with multiple images, which are stored within the HAM10000_metadata file. A total of 10,015 images from this dataset were utilized for skin cancer classification. From this dataset, a total of 25,331 images were utilized to classify skin cancer, which are publicly available on the internet.

### 5.3. Evaluation Metrics

**Accuracy:** The accuracy measure defines the instances that are classified correctly from the total number of instances in the classification algorithm, and it is formulated as

(25) $$Accuracy=\frac{\beta +\rho }{\beta +\rho +\eta +\omega }$$

where β implies true positives, η represents false positives, ω is the false negatives, and ρ denotes true negatives.

**Precision:** This metric is a measurement that denotes the proportion of true positives to false positives. It is denoted as follows:

(26) $$Precision=\frac{\beta }{\beta +\eta }$$

**Sensitivity:** This metric describes the true positive pixel’s proportion with respect to all the positive pixels, and it is mathematically denoted as

(27) $$Sensitivity=\frac{\beta }{\beta +\omega }$$

**Specificity:** This metric is known as the true negative rate, which summarizes the proportion of negatives with respect to negative pixels, and it is expressed as

(28) $$Specificity=\frac{\rho }{\rho +\omega }$$

### 5.4. Experimental Results

This section explores various image results obtained for the proposed DL-PCMNet model using the two datasets.

#### 5.4.1. Image Results Using ISIC 2019 Skin Lesion Dataset

**Figure 4:**
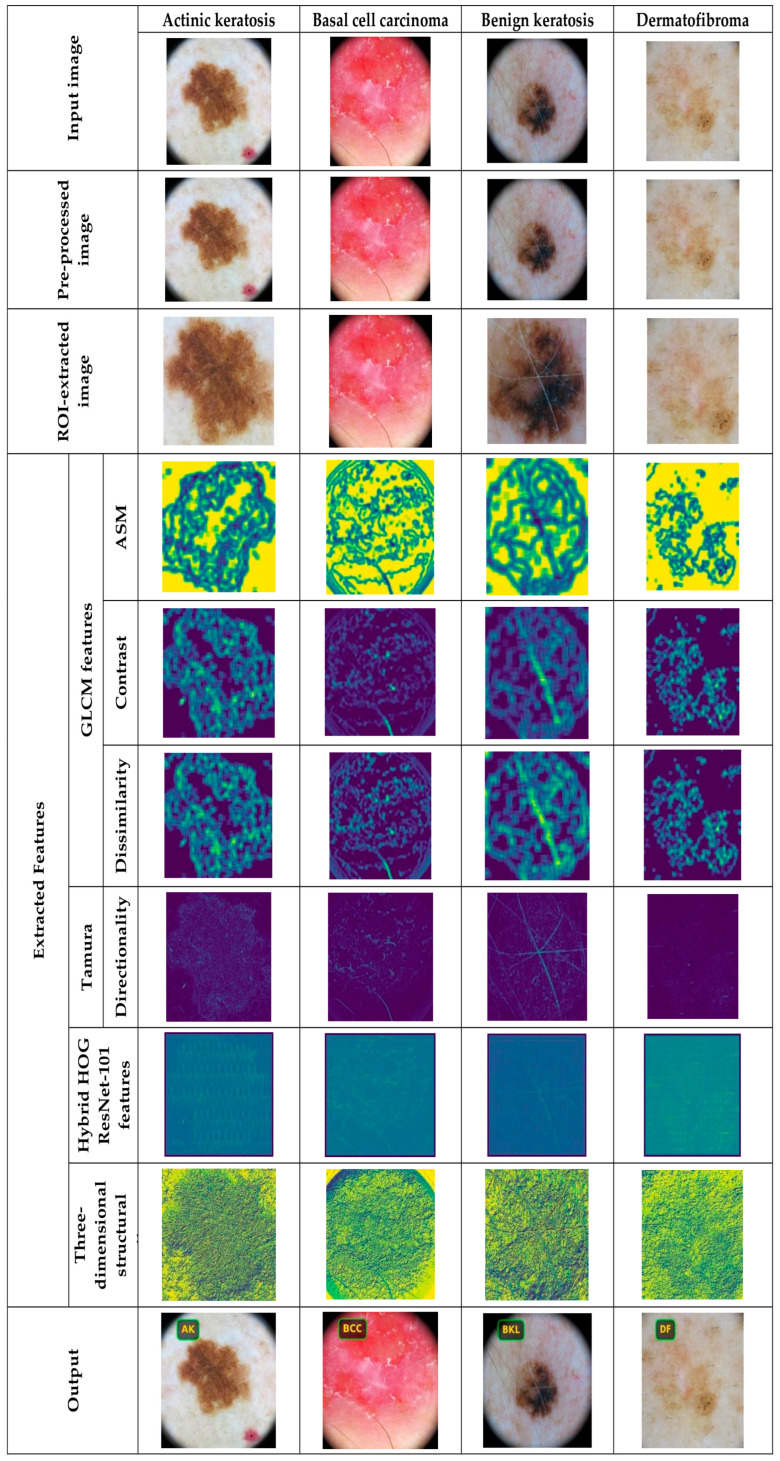
Image results of the proposed DL-PCMNet model.

**Figure 5:**
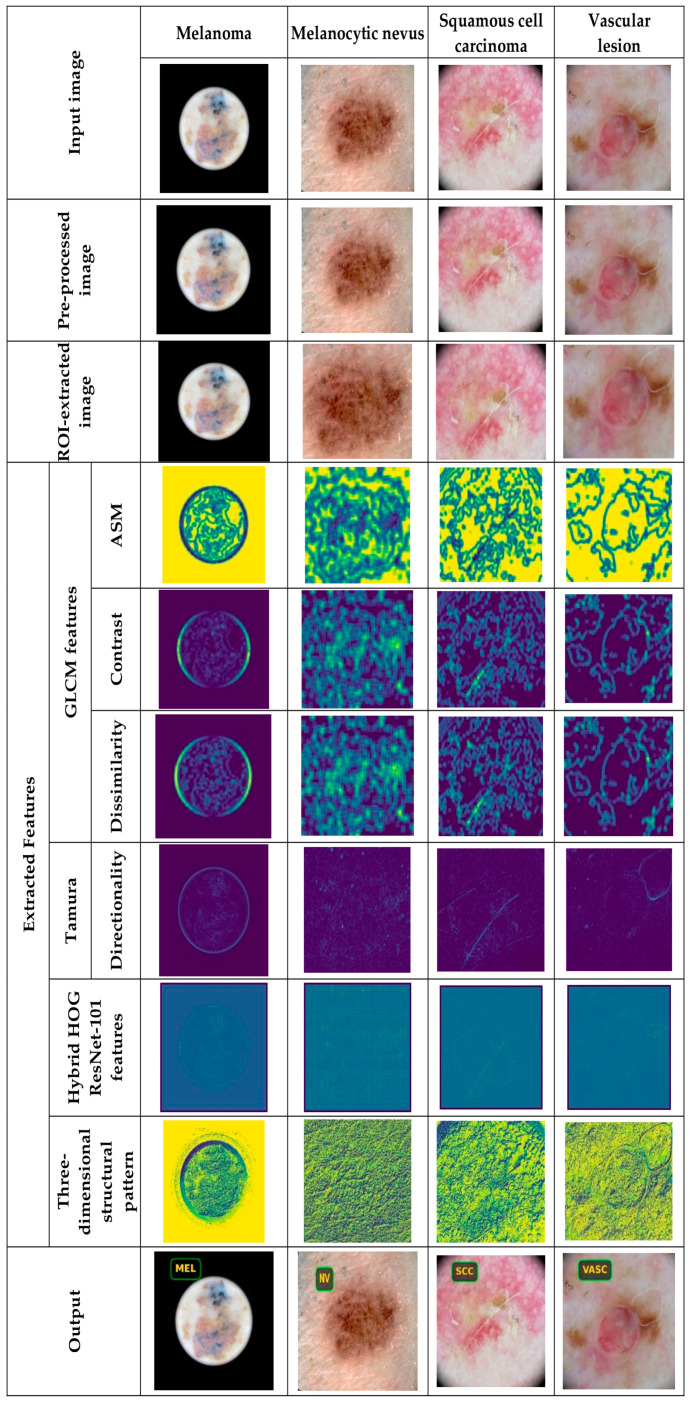
Sample results captured from the ISIC 2019 Skin Lesion dataset.

The proposed DL-PCMNet model’s image results from exploiting the ISIC 2019 Skin Lesion dataset are shown in Figure 4 and Figure 5. In Figure 4, the image results for classes such as basal cell carcinoma, benign keratosis, actinic keratosis, and dermatofibroma are depicted, and in Figure 5, classes like melanocytic nevus, squamous cell carcinoma, melanoma, and vascular lesions are visualized clearly.

#### 5.4.2. Image Results Using theSkin Cancer MNIST: HAM10000 Dataset

**Figure 6:**
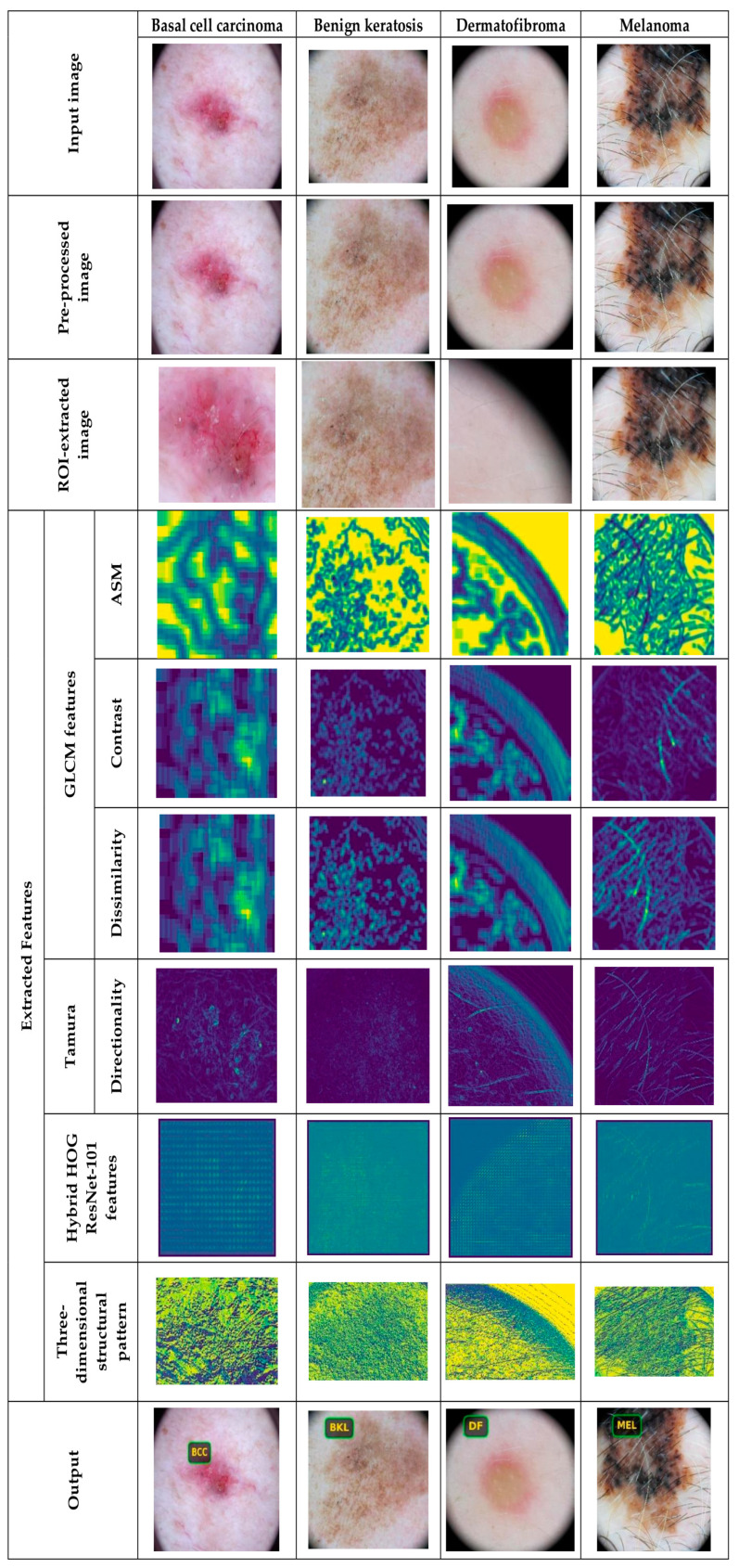
Image results using skin cancer MNIST: HAM10000 dataset.

**Figure 7:**
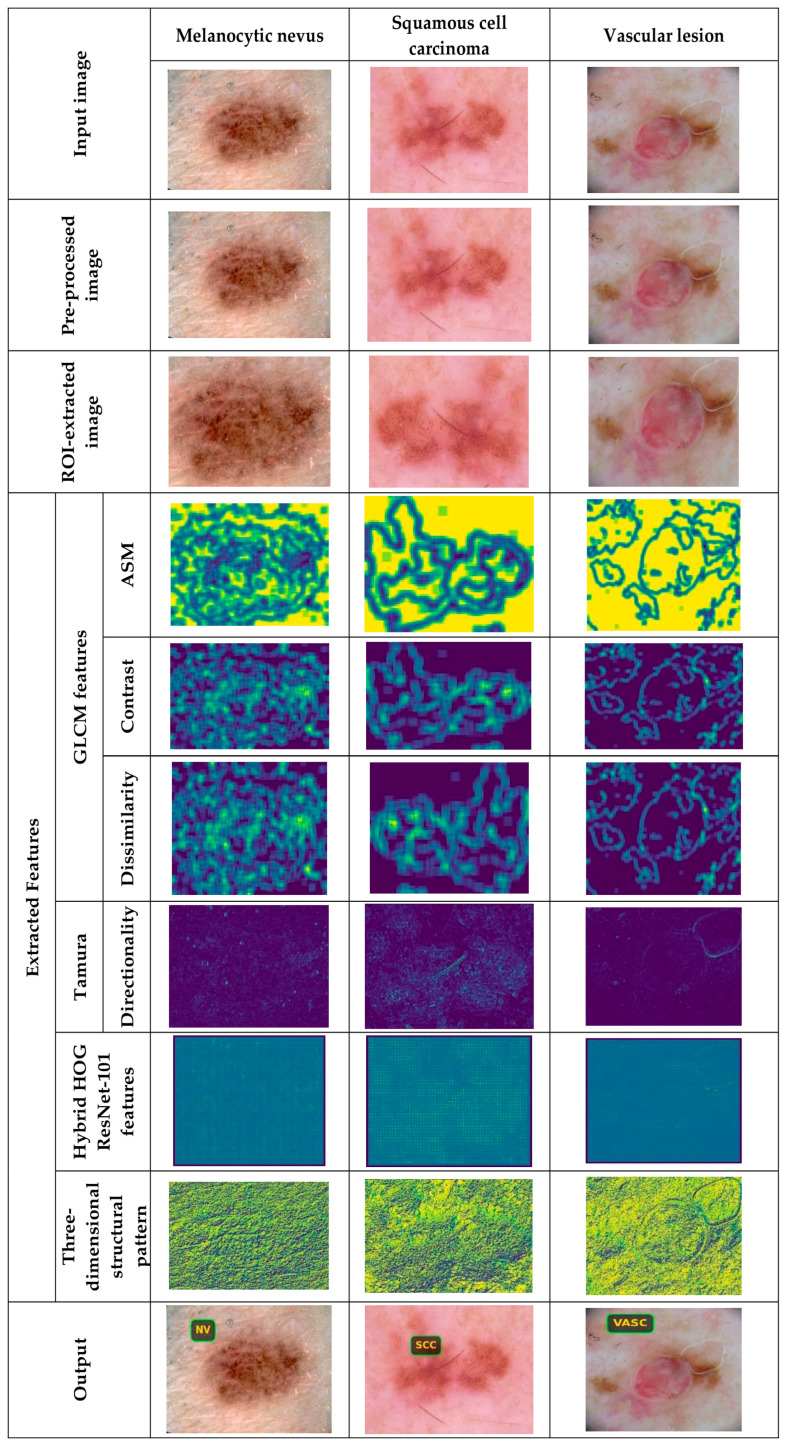
Sample results with skin cancer MNIST: HAM10000.

The image outcomes of the proposed DL-PCMNet model using the skin cancer MNIST: HAM10000 dataset are shown in Figure 6 and Figure 7. However, Figure 6 visualizes the results with classes like dermatofibroma, benign keratosis, basal cell carcinoma, and melanoma, whereas Figure 7 depicts the sample results with labels such as melanocytic nevus, squamous cell carcinoma, and vascular lesions, respectively.

### 5.5. Performance Analysis

The performance analysis of the proposed DL-PCMNet model using the ISIC 2019 Skin Lesion dataset and skin cancer MNIST: HAM10000 dataset is summarized in this section.

#### 5.5.1. Performance Analysis Using the ISIC 2019 Skin Lesion Dataset

**Figure 8:**
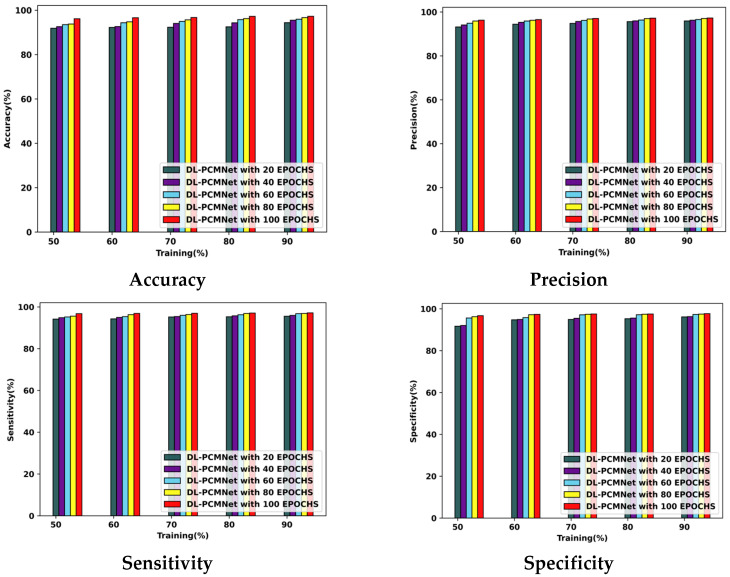
Performance analysis using the ISIC 2019 Skin Lesion dataset.

The DL-PCMNet model’s performance with the ISIC 2019 Skin Lesion dataset is visualized in Figure 8. The model acquired the following accuracy values with the 90% training data: 94.41% at epoch 20, 95.51% at epoch 40, 95.96% at epoch 60, 96.69% at epoch 80, and 97.28% at epoch 100. Furthermore, the DL-PCMNet model gained a precision of 95.92% at epoch 20, which later increased to 96.28% at epoch 40, 96.58% at epoch 60, 97.08% at epoch 80, and 97.30% at epoch 100. Likewise, the sensitivity of the DL-PCMNet model with training data of 90% is 97.17% at epoch 100, 96.86% at epoch 80, 96.76% at epoch 60, 95.96% at epoch 40, and 95.54% at epoch 20, respectively. The DL-PCMNet model’s specificity value at epochs 60, 80, and 100 is 97.32%, 97.50%, and 97.72% at 90% of training data.

#### 5.5.2. Performance Analysis Using Skin Cancer MNIST: HAM10000 Dataset

**Figure 9:**
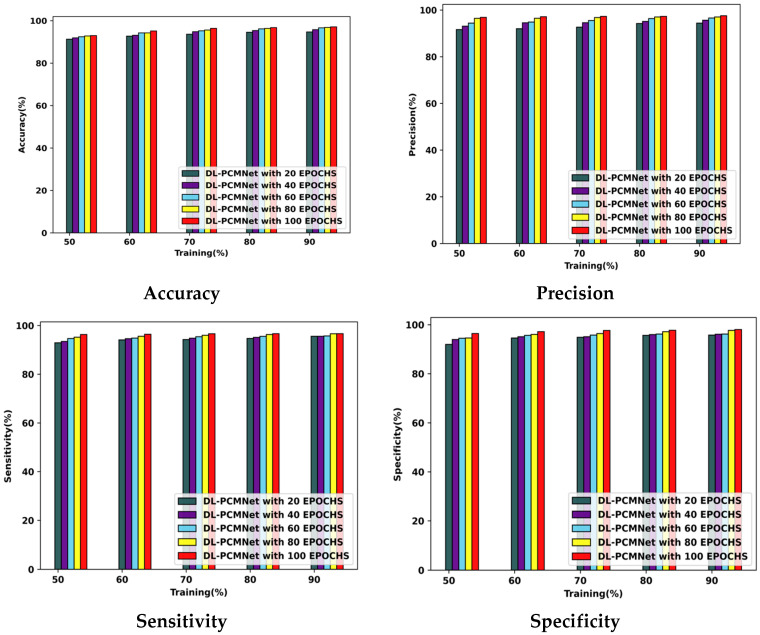
Performance analysis using skin cancer MNIST: HAM10000 dataset.

Figure 9 describes the DL-PCMNet model’s ability with training percentages and several varying epochs by employing the skin cancer MNIST: HAM10000 dataset. Here, the DL-PCMNet model’s accuracy value with the 90% training data is 94.74%, 95.81%, 96.68%, 96.83%, and 97.13% with epochs 20 to 100, respectively. The DL-PCMNet model achieved a precision of 94.38% at epoch 20, and further it increased to 95.66% at epoch 40, 96.57% at epoch 60. 97.03% at epoch 80, and 97.57% at epoch 100, respectively. The sensitivity value of the DL-PCMNet model at epochs 100, 80, 60, 40, and 20 is 96.67%, 96.63%, 95.73%, 95.60%, and 95.58% at 90% train data. Finally, the specificity of the DL-PCMNet model at 20 epochs is 95.77%, at 40 epochs is 96.12%, at 60 epochs is 96.21%, at epoch 80 is 97.69%, and at epoch 100 is 98.07%, respectively.

### 5.6. Comparative Methods

The performance of the proposed DL-PCMNet model is evaluated and compared with existing models such as hybrid VGG-SVM [1], Vision Transformer (ViT) [2], XAI-CNN [5], AlexGWO [6], Swin Transformer (ST) [7], CNN-LSTM [22], EfficientNet [27], DSCC_Net [28], and CNN [4] using the ISIC 2019 Skin Lesion image and Skin Cancer MNIST: HAM10000 datasets.

#### 5.6.1. Comparative Analysis Using the ISIC 2019 Skin Lesion Dataset

**Figure 10:**
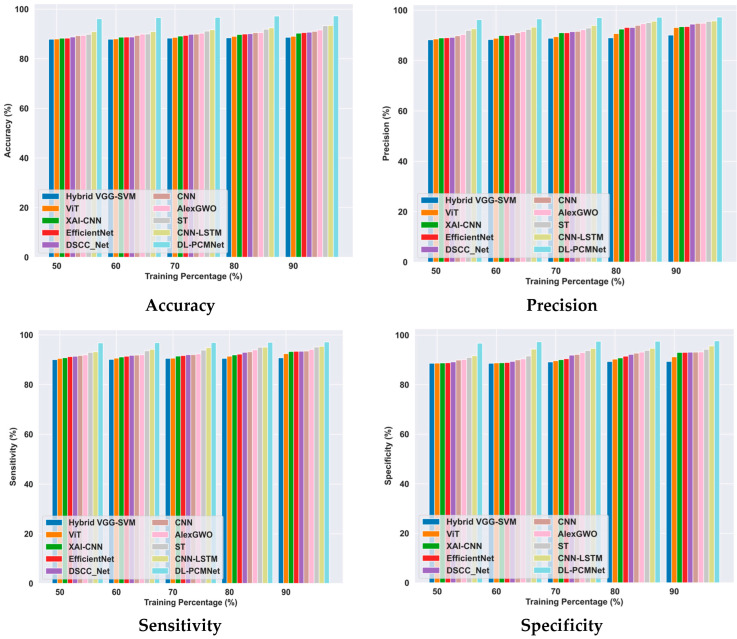
Comparative analysis using the ISIC 2019 Skin Lesion dataset.

Figure 10 illustrates the comparison of the DL-PCMNet model with other approaches using the ISIC 2019 Skin Lesion dataset based on training percentage. Here, the proposed DL-PCMNet model’s accuracy is 97.28%, with the 90% training data, an improvement over EfficientNet by 6.93%. The DL-PCMNet model’s value for precision is 97.30%, which is higher than hybrid VGG-SVM by 7.38%, ViT by 4.30%, XAI-CNN by 3.94%, AlexGWO by 2.53%, ST by 1.75%, and CNN-LSTM by 1.61%. The sensitivity of the DL-PCMNet model is 97.17%, which is greater than that of EfficientNet, with 3.89%. The specificity of the DL-PCMNet model is 97.72%, and it is higher than the hybrid VGG-SVM by 8.55%, ViT by 6.64%, XAI-CNN by 4.83%, AlexGWO by 4.59%, ST by 3.53%, and CNN-LSTM by 2.12%, respectively. Hence, the proposed model outperforms the other models with effective performance.

#### 5.6.2. Comparative Analysis Using the Skin Cancer MNIST: HAM10000 Dataset

**Figure 11:**
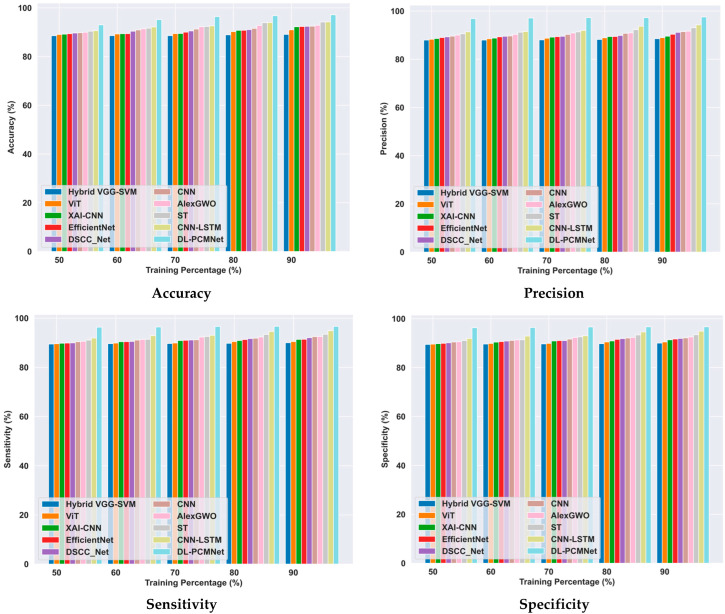
Comparative analysis using skin cancer MNIST: HAM10000 dataset.

The comparative analysis of the DL-PCMNet model is conducted by evaluating it against other existing models using the skin cancer MNIST: HAM10000 dataset, as visualized in Figure 11. The DL-PCMNet accuracy is 97.13%, which is 4.95% greater than the EfficientNet model. The precision value gained by the DL-PCMNet model is 97.57%, and it is 9.26% greater than the hybrid VGG-SVM, 8.82% greater than ViT, 8.21% greater than XAI-CNN, 6.06% greater than AlexGWO, 4.59% greater than ST, and 3.37% greater than CNN-LSTM, respectively. The DL-PCMNet model’s sensitivity is 96.67%, and it is higher than hybrid VGG-SVM by 6.89%, ViT by 6.41%, XAI-CNN by 5.52%, AlexGWO by 4.28%, ST by 3.34%, and CNN-LSTM by 1.85%. The specificity of the DL-PCMNet with the 90% training data is 96.67%, an improvement of 6.89% compared to the conventional hybrid VGG-SVM, 6.41% compared to ViT, 5.52% compared to XAI-CNN, 4.28% compared to AlexGWO, 3.34% compared to ST, and 1.85% compared to CNN-LSTM. Hence, the evaluation results show that the proposed model outperformed other existing models in terms of performance metrics.

### 5.7. Comparative Discussion

The existing models, such as hybrid VGG-SVM [1], Vision Transformer (ViT) [2], XAI-CNN [5], AlexGWO [6], Swin Transformer (ST) [7], CNN-LSTM [22], EfficientNet [27], DSCC_Net [28], and CNN, are evaluated with the proposed model to enhance the performance of the skin cancer classification. However, the presence of some issues makes them ineffective in accurate classification. The suggested hybrid VGG-SVM [1] method had high computational complexity issues and lacks applicability in real-world clinical applications. The ViT [2] model had poor performance, and it needs a vast amount of training data to improve its generalization capability. The model XAI-CNN [5] faces class imbalance issues, and the model fails to integrate attention mechanisms to achieve good performance. The suggested AlexGWO [6] model requires more dermoscopic images for training purposes, and its classification performance was low. The suggested ST [7] model fails to utilize advanced DL techniques, and it has limited interpretability in medical applications. Thus, these challenges are tackled by the proposed DL-PCMNet model by incorporating advanced feature extraction and distributed deep learning, which accurately classifies the type of skin cancer. The distributed learning technique solves the issues of computational complexity and speeds up the training process in an efficient manner. Furthermore, the utilization of a CNN and LSTM effectively enables the learning of complex and intricate patterns from the dermatoscopic images. Here, Table 3 depicts the comparative results of the DL-PCMNet model with several comparative approaches.

### 5.8. ROC Analysis

**Figure 12:**
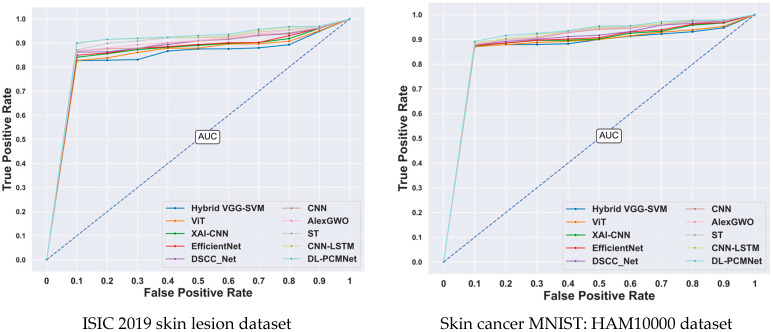
ROC analysis.

The Receiver Operating Characteristic (ROC) analysis of the DL-PCMNet model is illustrated in Figure 12. To analyze the sensitivity of the model, the ROC curve is plotted based on the True Positive Rate (TPR) and False Positive Rate (FPR), which are compared with other existing methods. The ROC of the proposed model reached a higher value of 1, whereas other traditional methods, such as Hybrid VGG-SVM, ViT, XAI-CNN, EfficientNet, DSCC_Net, CNN AlexGWO, ST, and CNN-LSTM, achieved 0.90, 0.92, 0.92, 0.92, 0.93, 0.94, 0.96, 0.97, and 0.97, respectively. Hence, the proposed DL-PCMNet model has high sensitivity, which helps to accurately identify skin cancer classes.

### 5.9. Time Complexity Analysis

**Figure 13:**
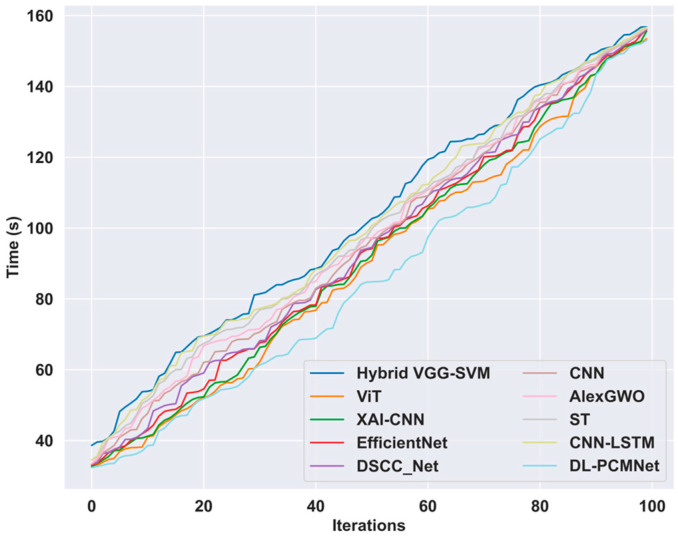
Time complexity analysis.

The computation time required for the proposed DL-PCMNet model in skin cancer classification is evaluated and compared with other existing methodologies across multiple iterations to showcase the performance of the model. The results highlight the computational ability because it consistently requires a significant reduction in time compared to other approaches. The utilization of distributed learning enhances the performance speed and reduces the computation time complexity. At iteration 100, the proposed model attained a minimum computing time of 152.98 s when compared to other existing approaches. An analysis of the DL-PCMNet model’s time complexity alongside existing methods is visualized in Figure 13.

### 5.10. Confusion Matrix

**Figure 14:**
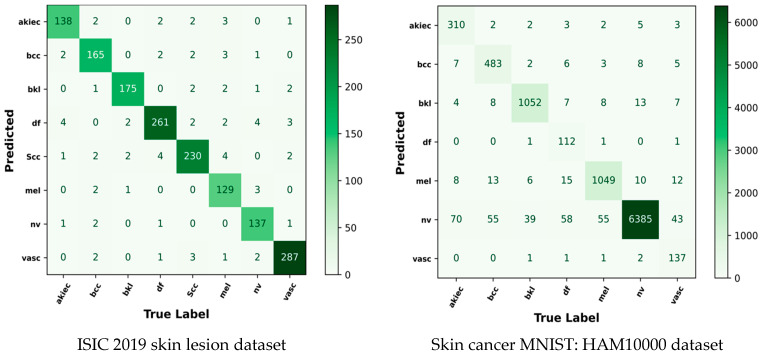
Confusion matrix analysis.

The confusion matrix is used to visualize and summarize the performance of the skin cancer classification model. It compares the predicted labels with the true labels of the dataset to identify the accurate and inaccurate classifications. The DL-PCMNet model classifies the input images into eight classes for the ISIC 2019 skin lesion dataset, and seven classes for the skin cancer MNIST: HAM10000 dataset. Here, the proposed model correctly predicted 138 as actinic keratosis, 165 as basal cell carcinoma, 175 as benign keratosis, 261 as dermatofibroma, 230 as melanoma, 129 as melanocytic nevus, 137 as squamous cell carcinoma, and 287 as vascular lesion. Consequently, the model also incorrectly identified very few images in each class. Hence, the proposed model effectively classifies skin cancer from the input dermatoscopic images. The confusion matrix of the DL-PCMNet model is shown in Figure 14.

### 5.11. Ablation Study for Feature Extraction Techniques

**Figure 15:**
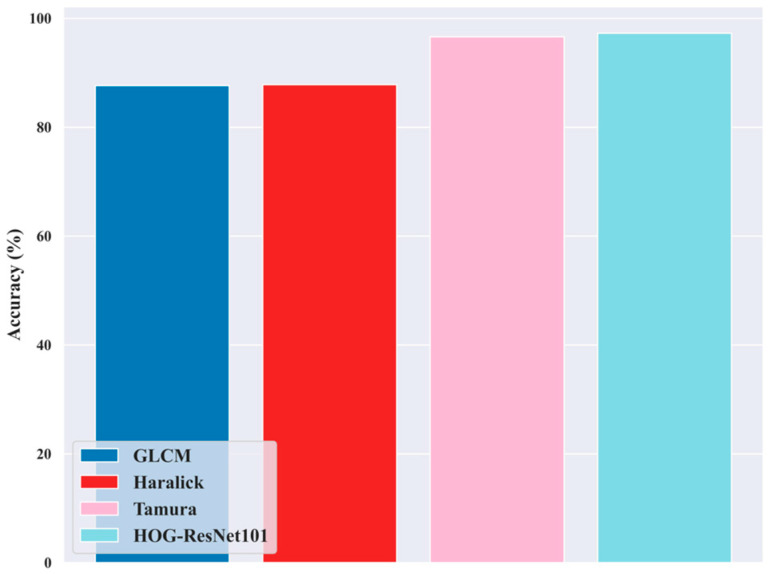
Ablation study.

Figure 15 illustrates the analysis of the Hybrid Haralick Tamura Gray-level Structured features approach utilized in the proposed DL-PCMNet model for the skin cancer classification process. The feature extraction methods extract meaningful and significant information from the input images. However, the feature extraction involves the GLCM features, Haralick features, Tamura features, Hybrid Hog ResNet-101 features, and a 3D-parallel structured pattern feature. The ablation study shows that these techniques attained high accuracy in terms of feature extraction, such as GLCM attained an accuracy of 87.64%, Haralick of 87.83%, Tamura of 96.61%, and HOG-ResNet101 of 97.28%. More specifically, the advantages of these feature extraction approaches are combined and utilized in this research to increase the model’s performance in skin cancer classification.

### 5.12. Statistical T-Test Analysis

The statistical significance test is conducted using the *t*-test analysis, which is tabulated in Table 4. The statistical *t*-test is analyzed based on the T-statistic and *p*-values. Here, the proposed DL-PCMNet model attained *p*-values less than 0.05, which indicates that the model is statistically significant.

## 6. Conclusions

Commonly, the deadliest form of cancer is skin cancer, which causes significant threat to individuals globally. Typically, these cancers are mainly caused by the abnormal growth of cells, and categorizing their types from dermoscopic images remains complex and challenging in the medical field. This research aims to analyze the existing issues occurring in the skin cancer classification, and it overcomes the challenges by integrating a distributed learning framework with a parallel CNN architecture. However, the distributed learning technique utilized in the DL-PCMNet model reduces the computational issues and prevents the risk of overfitting by increasing the ability of the learning process. Moreover, the model utilized a parallel CNN with an LSTM network to increase the accuracy and robustness by handling the complex data with spatial and temporal features. The proposed model shows better results over conventional methods using two datasets. The DL-PCMNet model achieved sensitivity, precision, accuracy, and specificity at 90% training data using the ISIC 2019 skin lesion dataset of 97.17%, 97.30%, 97.28%, and 97.72%, respectively. In the future, a hybrid optimization algorithm will be designed to train the hyperparameters of the model to enhance the accuracy and provide effective classification for skin cancer disease.

## Figures and Tables

**Table 1:** Texture features from GLCM.

GLCM Feature	Description	Formula	Dimension
**Homogeneity**	To calculate the rigidity of element distribution, homogeneity uses a value.	$F_{1}=\sum_{b,c=0}^{L-1}\frac{S\left(b,c\right)}{1+{\left(b-c\right)}^{2}}$ Where L $\mathrm{denotes}\;\mathrm{the}\;\mathrm{number}\;\mathrm{of}\;\mathrm{gray}\;\mathrm{levels}\;\mathrm{in}\;\mathrm{the}\;\mathrm{image}\;\mathrm{and}\;S\left(b,c\right)$ indicates the pixel of normalized probability with gray levels b and c.	$\left[N\times 256\times 256\times 1\right]$
**Contrast**	Contrast measures the difference in color of any object.	$F_{2}=\sum_{b,c=0}^{L-1}S_{b,c}{\left(b-c\right)}^{2}$	$\left[N\times 256\times 256\times 1\right]$
**Entropy**	The function entropy measures the randomness or complexity of an image.	$F_{3}=-\sum_{b=1}^{L}\sum_{c=1}^{L}S\left(b,c\right)\times \log\left\{S\left(b,c\right)\right\}$	$\left[N\times 256\times 256\times 1\right]$
**Dissimilarity**	Dissimilarity measures the dissimilarity of textures present in an image.	$F_{4}=\sum_{b,c=0}^{L}S_{b,c}\left|b-c\right|$	$\left[N\times 256\times 256\times 1\right]$
**Angular Second Moment**	It quantifies the uniformity of an image’s gray-level distribution.	$F_{5}=\sum_{b}\sum_{c}{\left(S_{b,c}\right)}^{2}$	$\left[N\times 256\times 256\times 1\right]$

**Table 2:** Hyperparameters.

Hyperparameters	Values
Batch size	32
Learning Rate	0.001
Number of epochs	500
Loss function	Categorical Cross-Entropy
Metrics	Accuracy
Activation Function	ReLU
Default Optimizer	Adam
Dropout rate	0.2
Number of convolutional layers	4
Number of LSTM layers	2
Filter size of convolutional layers	32, 8, 32, 8
Unit of LSTM layers	32, 8
Padding	Same
Pool size	2
Kernal size	3

**Table 3:** Comparative evaluation of the proposed DL-PCMNet model.

Techniques		Hybrid VGG-SVM	ViT	XAI-CNN	EfficientNet	DSCC_Net	CNN	AlexGWO	ST	CNN-LSTM	DL-PCMNet
ISIC 2019 skin lesion dataset	Accuracy (%)	88.64	89.04	90.28	90.54	90.70	91.05	91.60	93.33	93.39	97.28
	Precision (%)	90.11	93.11	93.46	93.52	94.48	94.79	94.83	95.59	95.73	97.30
	Sensitivity (%)	90.78	92.46	93.30	93.39	93.42	93.52	94.16	95.12	95.40	97.17
	Specificity (%)	89.36	91.22	93.00	93.02	93.12	93.13	93.23	94.27	95.65	97.72
Skin cancer MNIST: HAM10000 dataset	Accuracy (%)	89.06	90.98	92.22	92.33	92.41	92.46	92.85	94.17	94.25	97.13
	Precision (%)	88.53	88.96	89.56	90.37	91.16	91.47	91.65	93.09	94.27	97.57
	Sensitivity (%)	90.01	90.48	91.34	91.40	92.06	92.47	92.53	93.45	94.88	96.68
	Specificity (%)	90.01	90.48	91.34	91.69	91.92	92.16	92.53	93.45	94.88	96.68

**Table 4:** Statistical T-test analysis.

Methods/Metrics	ISIC 2019 Skin Lesion Dataset								Skin Cancer MNIST: HAM10000 Dataset							
	Accuracy		Precision		Sensitivity		Specificity		Accuracy		Precision		Sensitivity		Specificity	
	T-Statistic	*p*-Value	T-Statistic	*p*-Value	T-Statistic	*p*-Value	T-Statistic	*p*-Value	T-Statistic	*p*-Value	T-Statistic	*p*-Value	T-Statistic	*p*-Value	T-Statistic	*p*-Value
Hybrid VGG-SVM	2.34	0.08	1.99	0.12	2.57	0.06	2.44	0.07	1.69	0.17	1.69	0.17	2.65	0.06	2.65	0.06
ViT	2.41	0.07	1.92	0.13	1.63	0.18	2.20	0.09	2.05	0.11	3.07	0.04	2.66	0.06	2.66	0.06
XAI-CNN	2.67	0.06	2.70	0.05	2.09	0.10	1.97	0.12	1.77	0.15	2.74	0.05	3.31	0.03	3.31	0.03
EfficientNet	2.70	0.05	2.61	0.06	1.89	0.13	2.17	0.10	1.82	0.14	2.18	0.09	3.24	0.03	3.28	0.03
DSCC_Net	2.29	0.08	2.62	0.06	2.44	0.07	2.50	0.07	2.51	0.07	1.70	0.16	2.96	0.04	3.08	0.04
CNN	2.25	0.09	2.60	0.06	2.08	0.11	2.45	0.07	3.24	0.03	2.19	0.09	2.80	0.05	3.25	0.03
AlexGWO	2.55	0.06	2.73	0.05	1.96	0.12	2.62	0.06	3.49	0.03	2.59	0.06	3.32	0.03	3.32	0.03
ST	2.17	0.10	2.20	0.09	2.77	0.05	2.83	0.05	2.97	0.04	2.56	0.06	2.65	0.06	2.65	0.06
CNN-LSTM	2.01	0.12	2.59	0.06	3.44	0.03	3.78	0.02	3.18	0.03	1.97	0.12	2.79	0.05	2.79	0.05
DL-PCMNet	3.06	0.04	3.10	0.04	2.70	0.05	3.76	0.02	3.59	0.02	3.08	0.04	2.93	0.04	2.93	0.04

## Data Availability

The datasets adopted in the current study are publicly available online. The ISIC 2019 Skin Lesion images dataset is available at the link https://www.kaggle.com/datasets/salviohexia/isic-2019-skin-lesion-images-for-classification/data, accessed on 2 November 2025. The Skin Cancer MNIST: HAM10000 dataset is available at the link https://www.kaggle.com/datasets/kmader/skin-cancer-mnist-ham10000, accessed on 2 November 2025.
